# Recent Advances in Polysaccharides from *Cornus officinalis*: Extraction, Purification, Structural Features, and Bioactivities

**DOI:** 10.3390/foods14081415

**Published:** 2025-04-19

**Authors:** Shengfang Wang, Baotang Zhao, Xuemei Ma, Jing Zhang, Guofeng Li, Mingze Li, Qi Liang

**Affiliations:** 1 College of Food Science and Engineering, Gansu Agricultural University, Lanzhou 730070, China; m18735878759@163.com (S.W.); 18309409913@163.com (X.M.); 18230392709@163.com (J.Z.); guofengli82@163.com (G.L.); liangqi@gsau.edu.cn (Q.L.); 2 Institute of Agricultural Products Storage and Processing, Gansu Academy of Agricultural Sciences, Lanzhou 730070, China; lijun-68@163.com

**Keywords:** *Cornus officinalis*, polysaccharides, structure, activity

## Abstract

*Cornus officinalis*, as a medicinal plant, is rich in biologically active components, including polysaccharides, flavonoids, triterpenoids, and organic acids, which offer a variety of health benefits and significant potential for development in the food and pharmaceutical industries. *Cornus officinalis* polysaccharides (COPs) are considered among the primarily functional ingredients of the plant and are abundant in bioactivities. The present paper reviews the research conducted on the extraction, purification, structural properties, and biological activities of COPs. It also provides an overview of future development prospects, with a view to offering reference material for further development and research on COPs. In addition, the paper makes recommendations regarding theoretical preparations for the exploration of the application potential of COPs in the food industry and various other industrial fields.

## 1. Introduction

*Cornus officinalis*, also known as shu jujube, chicken feet, cornelian cherry, etc., is the dried mature fruit pulp of Cornus officinalis, a plant of the Cornaceae family, which has high edible and medicinal value [1] and is widely planted in Henan, Zhejiang, Shanxi, Anhui, Hunan, and Hubei provinces in China [2] (Table 1). It belongs to the Cornaceae family and can be consumed as an edible, functional food with tonic effects [3]. Its pulp can be used as a medicinal agent that tonifies the liver and kidneys while also acting as an astringent and aphrodisiac. The plant undergoes sequential flowering followed by leaf production. In autumn, it bears scarlet and red fruits that are aesthetically pleasing. The fruits are notable for their richness in active ingredients, including polyphenols, polysaccharides, flavonoids, saponins, vitamins, and minerals [4]. Among them, *Cornus officinalis* polysaccharides (COPs) are functional constituents of *Cornus officinalis*, with abundant physiological and pharmacological activities [5], for instance antioxidant, antimicrobial, antidiabetic, hypoglycemic, hypolipidemic, anti-inflammatory, and antitumor properties and immunomodulatory, nephroprotective, and neuroprotective activities [6,7,8]. At present, the bioactivity and functional application of COPs have also been a major hot topic.

The growing environment and management of *Cornus officinalis* has a significant effect on its polysaccharide synthesis, content, and bioactivity. *Cornus officinalis*, as a plant mainly cultivated in captivity, has an incomplete spontaneous growth. Wild *Cornus officinalis* resources have been reduced by overexploitation, and artificial cultivation has become the main way to conserve its resources and meet its medicinal and research needs. However, the dependence of artificial cultivation on a few high-quality varieties may lead to a reduction in genetic diversity, which in turn affects the structural diversity of polysaccharides. Artificial cultivation facilitates the standardization of the production process, which is essential for the quality control of polysaccharides, and the interference of heavy metal and pesticide residues on the purity of polysaccharides can be reduced through standardized cultivation.

Polysaccharides are biological macromolecules polymerized from monosaccharides, which are widely available in nature and possess a wide range of bioactive function [9,10]. In addition, the preparation of polysaccharide hydrocolloids [11], special foods [12], the development of food packaging materials [13], and the preparation of organic compounds (e.g., furfural) [14] have effectively tapped the potential resources of polysaccharides and enriched their application fields. It has been found that natural plant polysaccharides contain proteins, pigments, inorganic salts, and some fat-soluble impurities, which affect the purity, structural analysis, and functional properties of polysaccharides and eventually affect the actual mass and application value of the products. Consequently, the development of effective extraction methodologies is paramount for the study of polysaccharides. Polysaccharide extraction methods consist of some traditional extraction methods and emerging methods. However, commonly used methods include hot water extraction, ultrasound-assisted extraction (UAE), microwave-assisted extraction (MAE), and enzyme-assisted extraction (EAE). Polysaccharide purification methods are generally column chromatography, ion exchange chromatography, gel permeation chromatography, and affinity chromatography [15,16,17]. Polysaccharides can be separated according to their properties. However, established methods are associated with significant disadvantages. Consequently, intensive future research is needed to find the optimal process for extracting and purifying polysaccharides from plants [18]. The study of purified plant polysaccharide components can further elucidate the biological activities and functional mechanisms they possess. For Cornus officinalis, differences in varieties and fruit parts, growth environments, processing methods and storage methods may have an impact on the study of diverse COPs. In the current study, researchers have identified a wide range of bioactivities and health benefits of COPs and have studied their structure and conformational relationships in depth.

The idea of the homology of food and medicine was mentioned in *Huangdi Neijing Taisu*, the *Book of Eating*, and the *Treatise on Typhoid Miscellaneous Diseases*, which emphasized the connection and transformation between food and medicine, laying the foundation for the subsequent development of food therapy and medicine. The isolation and identification of natural plant polysaccharides from plants with the same origin as food and medicine have attracted a considerable amount of attention due to the wide application in food, medicine, cosmetics, and other fields. With the strategy of ‘Healthy China’ and the increasing awareness of public health care, the exploitation of safe, healthy, and green functional foods has a bright future [19]. Since 2023, when *Cornus officinalis* was added to the list of foods and herbs by the National Board of Health and Hygiene, it has also been developed as a tonic food, health drink, and dietary supplement [20]. Therefore, this paper examines the commonly used extraction and purification methods of COPs, describes their structural features and summarizes their bioactivities, and provides future perspectives on the structure and function, development, and application of COPs.

## 2. Extraction and Purification

### 2.1. Extraction

The extraction method is pivotal in determining the quantity, structure, and biological activity of natural plant polysaccharides. Currently, besides conventional methods (Table 2), UAE, MAE, and EAE methods are among the most common techniques used to disrupt the cell wall structure and expedite solvent entry into the cell interior for efficient extraction. The mentioned methods employ the cavitation effect of ultrasonic, the energy effect of microwave, and the specific hydrolysis of enzymes, respectively. Ultrasonic cavitation and microwave radiation can change the structure of polysaccharides. Consequently, it is essential to control the intensity and time of ultrasonic and microwave energy in order to minimize the damage of polysaccharides. Moreover, MAE can only be used for the extraction of heat-stable materials, and heat sensitive materials can be denatured or inactivated. EAE is green, which can more effectively preserve the structure of polysaccharides, but it is costly and susceptible to the effects of temperature, pH, metal ions, etc. [21,22]. You et al. extracted the polysaccharides from *Cornus officinalis* fruits by using the EAE method. Based on a one-way experimental design, the RSM method was used to estimate and optimize the experimental variables. The outcomes demonstrated that the optimum yield of COPs was 9.29 ± 0.31%, the COP yield of EAE was superior to that of UAE, and the cost of equipment was lower than that of UAE [23].

**Figure 1:**
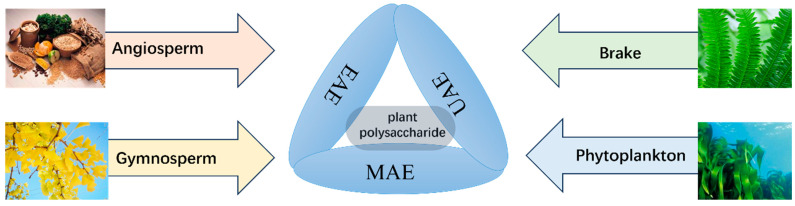
Preparation of plant polysaccharides by auxiliary extraction methods.

In summary, the selection and coordination between various extraction methods can increase the yield of plant polysaccharides (Figure 1). While the combination technology of some emerging auxiliary extraction methods of polysaccharides needs to be further explored, researched, and improved, there are few studies on the extraction methods of *Cornus officinalis*.

#### 2.1.1. Supercritical Fluid Extraction (SFE)

SFE is a method that uses supercritical fluids as solvents to extract certain effective components from solids or liquids and to separate them. It is characterized by easy operation, fast extraction speed, high selectivity, and environmental protection. Supercritical carbon dioxide diffusion rate is high and easy to adjust the solvent strength, and analyte solvent-free recovery is simple, but the main shortcoming is the low polarity [28]. Appropriate entrainers or modifiers (ethanol, water, etc.) can be used to increase its solubility and selectivity for the target analytes to improve the target yield. In addition, the difficulty in controlling SFE parameters, operational complexity, and high equipment requirements restrict the widespread use of SFE technology [29]. The plant polysaccharides that have been studied by the SFE method include polysaccharides taken from the leaves of bamboo, polysaccharides from ashwagandha flowers, and polysaccharides from deciduous ginkgo, etc. This technique has been less studied for the extraction of plant polysaccharides, and no report has been made about the extraction of polysaccharides from *Cornus officinalis*.

#### 2.1.2. Aqueous Two-Phase Extraction (ATPE)

ATPE is a technique for achieving separation based on the difference in partition coefficients of substances between different phases. When two polymers or a polymer and an inorganic salt are mixed at an appropriate concentration, two phases that are immiscible with each other are formed. These substances have different solubilities in the different phases, which results in their uneven distribution in the two phases [30,31]. By exploiting this difference in distribution, the separation and purification of substances can be achieved. Two commonly used substances are polyethylene glycol/dextran and polyethylene glycol/salt, whose polymer-salt biphasic system has the advantages of good biocompatibility, low cost, and mild operating conditions. Yet, the disadvantages of the formation of polymers are that they are easy to emulsify, and they have high viscosity and a high cost, which limits the industrial production and application of the technology to a certain extent [22,32,33]. However, ATPE can achieve extraction and separation in a single step, so the target components are not repeatedly purified. Due to the advantages of having a high yield, being environmentally friendly, having easy amplification, low cost, less harm to molecular biological activity, and so on, it can be extended gradually from the separation of biological molecules to the extraction of small molecules. ATPE has been extensively used in the extraction of polysaccharides from *Lycium chinensis*, *Schisandra chinensis*, and fennel [19], whereas the study of *Cornus sativus* polysaccharides needs to be further explored.

#### 2.1.3. Microwave-Assisted Aqueous Two-Phase Extraction (MATPE)

MATPE is a novel extraction method combining microwave extraction and double aqueous phase extraction, which has the advantages of being rapid and efficient, having uniform and selective heating, and it can also obtain high-purity polysaccharides, which can effectively avoid subsequent polysaccharide deproteinization and pigmentation operations. Xiao et al. optimized the process of extracting COPs using MATPE with 300 W microwave power, a 35% ethanol volume fraction, 22% ammonium sulphate, and a material–liquid ratio of 1:20 (g/mL) and obtained a yield of COPs of (12.04 ± 0.17)% [34]. Currently, it is necessary to study the MATPE of COPs and the purification of the resulting polysaccharide crude extracts to provide a reference basis for the technology of obtaining plant polysaccharides

#### 2.1.4. Ultrasonic-Assisted Aqueous Two-Phase Extraction (UAATPE)

The method of combining UAE and ATPE, that is, UAATPE, allows COPs to be extracted and purified in one step. Tan et al. obtained the highest yield of COPs (7.85 ± 0.09)% under 350 W ultrasound power, a 51 °C extraction temperature, a 17 mL/g liquid/feed ratio, and 38 min of extraction time, and there is no literature report on the study of UAATP of COPs [29]. This method increases extraction efficiency and improves the quality of the extract by disrupting cell walls or other barriers, making it easier to obtain the target substance. In order to further improve its extraction efficiency and effectiveness, UAATPE is often used in conjunction with other technologies, and the application of this technology from the initial more common plant active ingredients, such as tea polyphenols, flavonoids, and so on, to gradually expand it to more types of substances and raw materials in the future, related to the extraction equipment, is expected to achieve intelligent and automated control.

#### 2.1.5. Ultrasonic Enhanced Compound Enzyme Auxiliary Extraction (UCEA)

The ultrasound-enhanced composite enzyme-assisted extraction method is a process that utilizes ultrasound technology to facilitate the extraction process by means of composite enzymes. The primary objective of this method is to enhance the efficiency of the extraction process and concurrently reduce the associated production costs. The application of this method is predominantly in the extraction of active ingredients from natural products, through the formation of a multi-frequency composite ultrasound cavitation field to expand the scope of action, increase the effective action space, and enhance the cavitation effect, thereby accelerating the rapid dissolution of natural products’ active ingredients. You et al. carried out the extraction optimization of ultrasound-enhanced composite EAE of polysaccharides of *Cornus sativus*, which was achieved with an extraction temperature of 49.6 °C, ultrasound time of 40.41 min, and ultrasonic power of 308.07 W. The experimental yield of the resulting polysaccharide was 11.02 ± 0.41% [35]. UCEA can be utilized for the extraction of plant proteins, natural colors, and flavors.

#### 2.1.6. Ultrasonic-Microwave Synergistic Extraction (UMSE)

UMSE is a complementary technique developed from the ultrasonic and microwave methods, which overcomes the deficiencies of the two techniques, achieves rapid, homogeneous, and low-temperature extraction, and has a good potential for the modification and application of polysaccharides in the functional food industry [36]. For example, Yin X et al. showed that the optimum process conditions for obtaining COPs by UMSE were an extraction time of about 31 min, microwave power of about 99 W, and a water-to-feedstock ratio of about 28, resulting in a COP yield of 11.38 ± 0.31% [37]. For the UMSE and purification of polysaccharides, the technology needs to be developed and improved.

### 2.2. Purification

Purification requires a combination of techniques for separation and purification, for example, ethanol precipitation, dialysis, deproteinization, decolorization, chromatography, etc. [38]. The most common methods used to remove proteins from crude extracts are Sevag, TCA, and enzymatic methods, as well as HCl, NaCl, and CaCl_2_ methods [39]. Colors are removed using organic reagents such as ethanol, petroleum ether, or by using resins, activated carbon, polyacrylamide, or hydrogen peroxide [40,41]. These impurities adversely affect the structural modelling of polysaccharides and the study of their biological activities. They may interfere with the analytical process and lead to inaccurate experimental data, which in turn limit the in-depth understanding of the polysaccharide properties and functional investigations. After the above removal treatments, polysaccharides are still mixtures with heterogeneity in chemical composition, degree of polymerization, molecular shapes, etc. To obtain a single-polysaccharide pure product, the mixture needs to be subjected to graded purification. The most commonly used ion exchange columns are DEAE-Sepharose Fast Flow [42], DEAE-cellulose column [43], DEAE-52, etc., and the most commonly used gel columns are Sephadex G-100 [44], Sephadex G-75, Sephadex CL-6B, etc. According to previous research, separation and purification can destroy the structure of polysaccharides, and large molecules are cleaved into small molecules overflowing from the cell membrane, which makes the molecular mass of polysaccharides show a tendency to decrease after purification [45]. However, it can ensure the accuracy of the polysaccharide structural resolution and yield high-purity polysaccharides on the basis of retaining the original structure.

**Figure 2:**
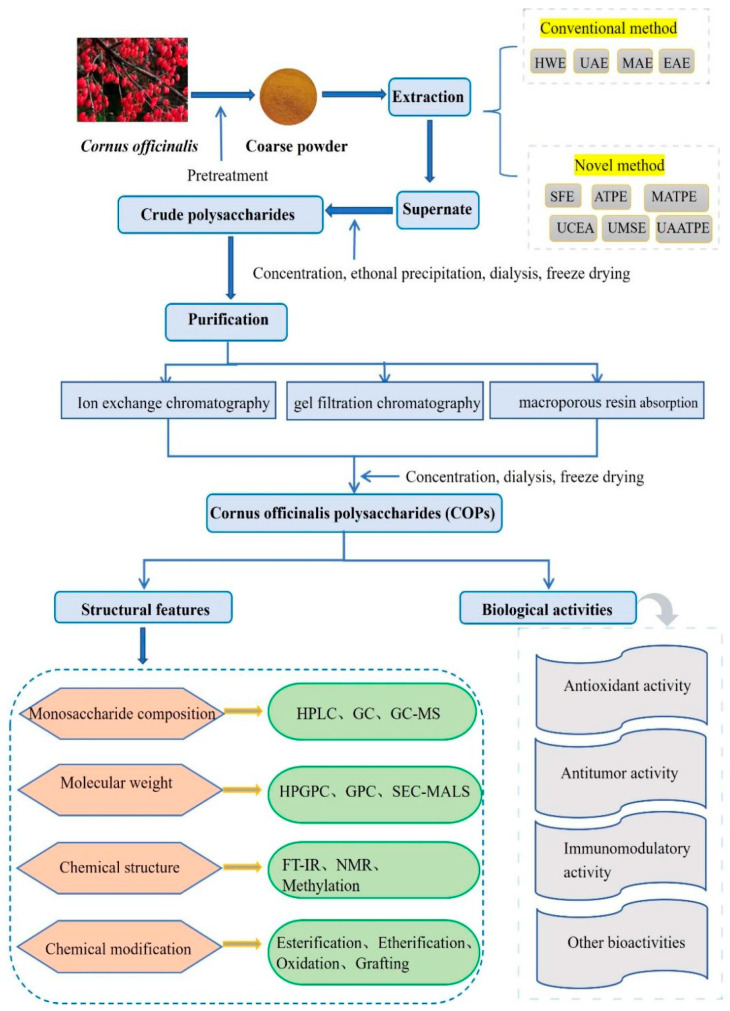
Schematic diagram of the commonly used research methods for COPs.

In conclusion, the *Cornus officinalis* polysaccharide fraction was obtained after ethanol precipitation of the *Cornus officinalis* extract, deproteinization by the Sevag method, and decolorization by hydrogen peroxide, through appropriate chromatographic methods, the removal of small molecule impurities by dialysis or ultrafiltration, evaporation, precipitation with ethanol, filtration, and lyophilization. The overall technology roadmap for Cornus polysaccharides is shown in Figure 2.

## 3. Structural Features and Physicochemical Properties

### 3.1. Monosaccharide Composition

In the study of the chemical composition of polysaccharides, the content and proportion of monosaccharides play a crucial role. Not only are these monosaccharides the building blocks for the formation of the complex structure of polysaccharides, but their type and quantity directly affect the interactions between polysaccharide molecules. A full understanding of the biological activity of polysaccharides requires knowledge and control of the composition of these monosaccharides. At the same time, these chemical structures are closely related to the digestion and absorption, metabolism, and potential biological effects of polysaccharides in the human body [46]. The monosaccharide composition was mainly assayed by methylation, acid hydrolysis, high-performance liquid chromatography (HPLC), gas chromatography (GC), high-performance gel permeation chromatography (HPGPC), and gas chromatography-mass spectrometry (GC-MS) [47]. It was found that COPs were rich in variety and varied in monosaccharide composition. In their study, Yin et al. optimized the extraction process and separated five purified fractions: COP, COP1, COP2, COP3, and COP4 by sequential purification using DEAE-52 and Sephadex G-100 chromatography, which were comprehensively analyzed and identified as a complex polysaccharide consisting mainly of Glc, Ara, Fuc, Xyl, Man, and Rha, with COP3 and COP4 being free of Rha [37]. This may be due to material site and properties as well as experimental errors affecting the monosaccharide composition of the fractions. The study by Tan et al. using ultrasound-assisted dual-phase aqueous extraction showed that the obtained *Cornus officinalis* polysaccharide fraction COPs-4-SG consisted of GalA, Ara, Man, Glc, and Gal in a molar ratio of 34.82:14.19:6.75:13.48:12.26. COPs-4-SG is a heteropolysaccharide with a different chemical composition and a non-triple helical structure [29]. Yang et al. isolated the water-soluble polysaccharide FCAP1 from the alkaline extract of *Cornus officinalis* fruits, with a monosaccharide composition of Fuc, Ara, Xyl, Man, Glc, and Gal, with a molar ratio of 0.29:0.19:1.74:1:3.30:1.10, respectively [6]. It was found that the difference in the composition of the prepared monosaccharides of *Cornus sativus* might be attributable to variations in the raw materials and the preparation process.

### 3.2. Average Molecular Weight (Mw)

Mw is one of the structural index of polysaccharides which can influence their physicochemical and biological properties [48]. Too small a molecular mass of polysaccharides makes it difficult to form an active space structure, and too large a molecular mass results in too large a polysaccharide molecule, which is also unfavorable for polysaccharides to cross the cell membrane and enter the organism to play their roles [45]. The molecular weight of polysaccharides affects the biological activity and structure of the organism, while the source of the plant and the extraction process also influence the molecular weight of the polysaccharides significantly [49]. The average molecular weight can be determined by various methods, such as high-performance gel exclusion chromatography (HPSEC), high-performance liquid chromatography (HPLC), high-performance gel permeation chromatography (HPGPC), etc., and the development of some multi-detector technology has improved the accuracy of molecular weight determination and expanded the scope of application. Similarly, a new thought for the research on polysaccharide structure has been provided by the application of ionic liquids in molecular weight characterization [50]. Regarding the determination of molecular weight of other plant polysaccharides, APFC-2 was determined to be a homogeneous polysaccharide with a molecular weight of 63.0 kDa using high-performance liquid chromatography [51]. Artemisia absinthium polysaccharide’s (AAP) average molecular weight of 16 kDa was determined using the GPC-MALLS-RI system [52]. Zeng et al. determined the chemical composition ratio, monosaccharide ratio, triple-helix conformation and in vitro antioxidant activity of LBP polysaccharides of varying molecular weights. The findings demonstrated that there was no statistically significant difference in the total sugar content of LBPs with different molecular weights; however, there were significant differences in the total protein content and total polyphenol content. The proportion of glucose was found to be dominant in the low-Mw polysaccharides, whereas it was replaced by arabinose and galactose in the high-Mw LPs. It was further demonstrated that LBPs had no detectable effect on the glycosidic bond arrangement or major functional groups, and the effect on the triple-helix conformation might only be present in substances with molecular weights greater than 3 kDa. The various antioxidant activities are influenced by multiple interacting factors, including molecular weight, monosaccharide composition ratio, chemical composition, and chemical reaction mechanism [53]. Mw can help to reveal the intrinsic connection between polysaccharide structure and function, deepen the understanding of the mechanism of polysaccharide biological action, help to track the synthesis, modification, and degradation of polysaccharide in organisms, understand the changing law of polysaccharide molecular weight at different stages, and provide important information for exploring the regulatory mechanism of polysaccharide biosynthesis and metabolic network.

### 3.3. Chemical Structures

FT-IR techniques are frequently employed in the identification of functional groups, glycoconjugates, and sugar residues found in polysaccharides [54]. By means of Smith’s degradation, periodate oxidation, methylation, GC-MS, NMR, and acid hydrolysis, the types and locations of glycosidic bonds can be determined, which allows the analysis of monosaccharides and the order of glycosidic bonding [55,56], attachment sites and substitution of functional groups, and the deduction of polysaccharides from their backbone, branched chains, and repeating units. Bai et al. found by analysis that APFC-2 was a low-methyl esterified pectin polysaccharide characterized by a backbone of T-β-Galp-(1 → 6)-β-Galp-(1 → 3,6)-β-Galp-(1 → [4)-α-GalpA-OMe-(1 → 4)-α-GalpA-(1 →]m → [2,4)-α-Rhap-(1 → 4)-α-GalpA-(1 →]n and branching constitutes T-Araf-(1 →, →3)-α-Araf-(1 →, → 3,5)-α-Araf-(1 →, and → 5)-α-Araf-(1 → [51]. Yang et al. found that FCAP1 was a highly branched xyloglucan with a backbone consisting of β-(1 → 4)-linked glucose residues. The main chain was partially substituted at the O-6 position of the glucosyl residue by Xylp-(1 →), Galp-(1 → 2)-Xylp-(1 →) and Fucp-(1 → 2)-Galp-(1 → 2)-Xylp-(1 →). This branch also contained → 3)-Ara-(1 → 4)-Glc-(1 → 4)-Man(1 → 4,6)-Man-(1 →), Ara-(1 →), and Gal-(1 →) [6]. Fu et al. revealed that PFC-3 was mainly composed of 1,3-α-D-Xylp, 1,6-α-D-Galp, 1,2-α-D-Glcp, and T-α-D-Galp, with backbone fragments containing →6)-α-D-Galp-(1 → 2)-α-D-Glcp-(1 → 3)-α-D-Xylp-(1 →) [57]. The above structural fragments of *Cornus officinalis* polysaccharides are presented in Table 3.

### 3.4. Chemical Modification

Methods of modification of polysaccharide include physical modification [58], chemical modification [59], and biological modification [60]. Biological modification is mainly applied to microbial polysaccharides. The modification methods of plant polysaccharides are mainly chemical modification and physical modification, and the current modification methods are mainly the chemical modification of plant polysaccharides [61,62], that is, the chemical alteration of the structure of purified crude polysaccharides to produce more bioactive or novel biologically active polysaccharide derivative [63]. Basic chemical modification methods of polysaccharides are selenylation, sulfonation, acetylation, carboxymethylation, and phosphorylation [62]. Chemical modification replaces the hydroxyl group of the original polysaccharide with an objective group, for instance, a sulfate, carboxymethyl, acetyl, selenate, or phosphate group, and the composition, molecular weight, and morphological characteristics of the monosaccharide are changed by the above chemical modifications [19]. Substituents and changes in spatial structure, which increase water solubility and the activity of functional groups, enhance the bioactivity of specific biological functions [64]. NMR is extensively employed for the structural interpretation of polysaccharides and their derived products and can also confirm the results of polysaccharide modifications; each modification method has its own features. For instance, sulphation improves antioxidant activity and immunomodulation, carboxymethylation improves water solubility and biological activity, and acetylation improves emulsification. Consequently, appropriate modification methods can be selected for different needs to modify plant polysaccharides appropriately [22,59]. Although chemical modification studies for *Cornus officinalis* polysaccharides have not been reported, modification studies for other plant polysaccharides have been applied (Table 4).

## 4. Biological Activities

### 4.1. Antioxidant Activity

**Figure 3:**
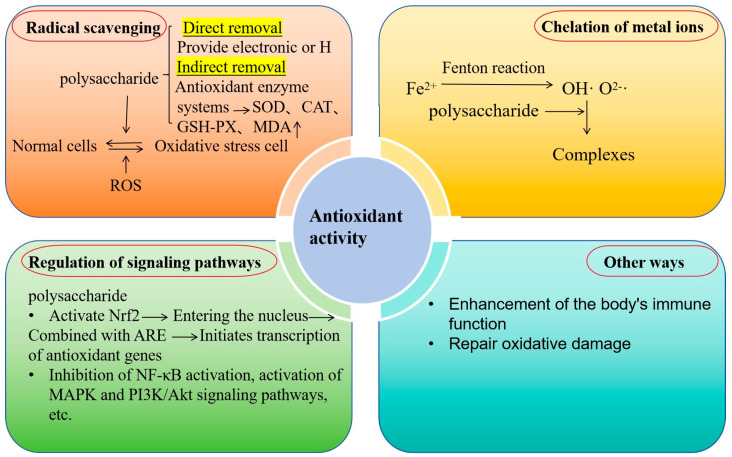
Antioxidant mechanism of polysaccharide molecules.

Antioxidants include in vivo and in vitro antioxidants. In vitro antioxidant activity is mainly characterized by hydroxyl radical scavenging, removal of superoxide anion, restoring power, and ant lipid peroxidation. In vivo antioxidant activity is mainly determined by total antioxidant abilities [85]. The antioxidant mechanism in the body is shown in Figure 3. Free radical production is known to regulate cell growth and inhibit the propagation of bacteria and viruses. Conversely, too many free radicals can cause damage to DNA and proteins, affecting cellular function and causing cancer, inflammation, aging, and cardiovascular diseases [86]. Bioactive polysaccharides from various natural sources are important in the elimination of free radicals, which may be used as novel antioxidants [87]. Li et al. investigated and compared the antioxidant activity of two isolated polysaccharides, which showed potential antioxidant activity by scavenging superoxide anion (O_2_^−∙^) and hydroxyl radical (OH∙), and found that the antioxidant activity of PFCC I was higher than that of PFCA III [88]. Despite the surge in research interest in polysaccharides, the correlation between polysaccharide antioxidant activity and chemical properties such as monosaccharides, molecular weight, and chemical structures remains to be elucidated. To advance the development of polysaccharides as a natural antioxidant, it is imperative to ascertain the relationship between their antioxidant activity [89].

### 4.2. Antitumor Activity

**Figure 4:**
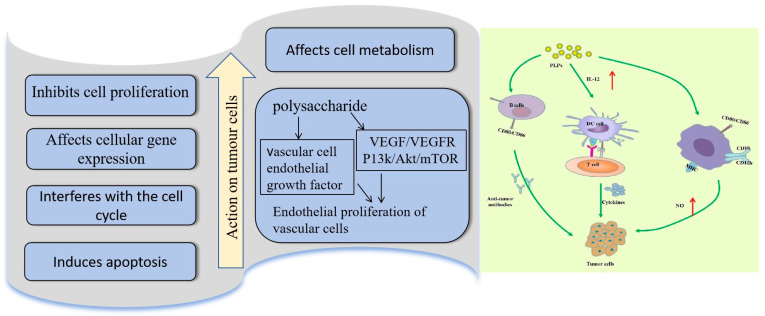
Antitumor mechanism of polysaccharide molecules [93].

Polysaccharides have been identified as a potential immunotherapy, with the capacity to stimulate the immune response of tumor cells and augment their immune surveillance capability [90]. Plant polysaccharides can inhibit tumor proliferation and growth by inhibiting tumor cell invasion and metastasis, inducing apoptosis, influencing the cell cycle, and regulating the tumor microenvironment [91]. The antitumor effects of polysaccharides are shown in Figure 4. Zou et al. used the S180 sarcoma mouse model as their research object to observe the ex vivo and in vivo antitumor effects of COPs and their modulatory effects on CD4+ T cells, CD8+ T cells, interleukin IL-2, and interleukin IL-4 in the peripheral blood of the loaded tumor-bearing mice and compared them with those of the negative control group. The results indicated that the polysaccharides of *Cornus officinalis* exhibited a substantial tumor inhibitory effect on S180 (*p* < 0.01), with the potential to enhance the expression of peripheral blood CD4+ T cells, decrease the expression of CD8+ T cells, increase the level of interleukin IL-2, and reduce the level of interleukin IL-4. Additionally, a positive correlation was observed between the dose and the concentration of polysaccharides and their effect on tumor inhibition. Thus, COPs exerted antitumor effects by modulating the abnormal immune status of hormonal mice [92].

### 4.3. Immunomodulatory Activity

**Figure 5:**
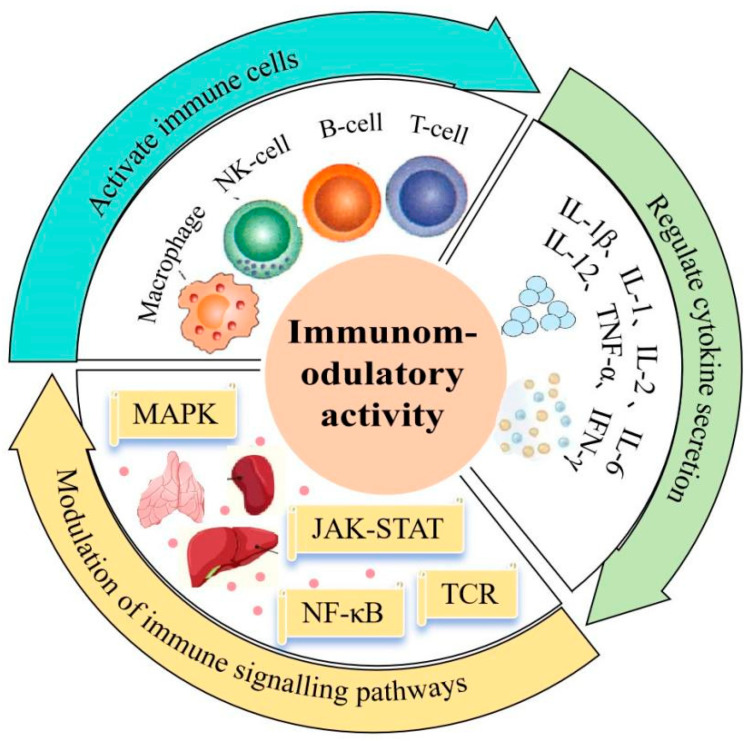
Immunomodulatory mechanisms of polysaccharides.

Plant polysaccharides with multifunctionality are known as biological response modifiers (BRMs), which not only activate immune cells, including T cells, B lymphocytes, macrophages, and natural killing cells but also activate the body and promote the production of cytokines, so as to show the regulating role of the immune system in various ways [94] (Figure 5). Shi et al. studied the effects of polysaccharide of Fructus Corin (PFC) on the immune function of cyclophosphamide (CTX)-induced immune function and its related mechanisms and found that PFC could enhance the immune function of CTX-induced immunocompromised mice, and the specific mechanisms may be related to the regulation of the intestinal flora and the Th1/Th2 ratio [95]. Du et al. discovered that crude and processed COPs had potentiating effects on non-specific immunity, specific body fluid immunity and specific cell immunity in immunosuppressed mice, and that the effects of polysaccharides were significantly increased by processing with wine [96].

### 4.4. Other Bioactivities

**Figure 6:**
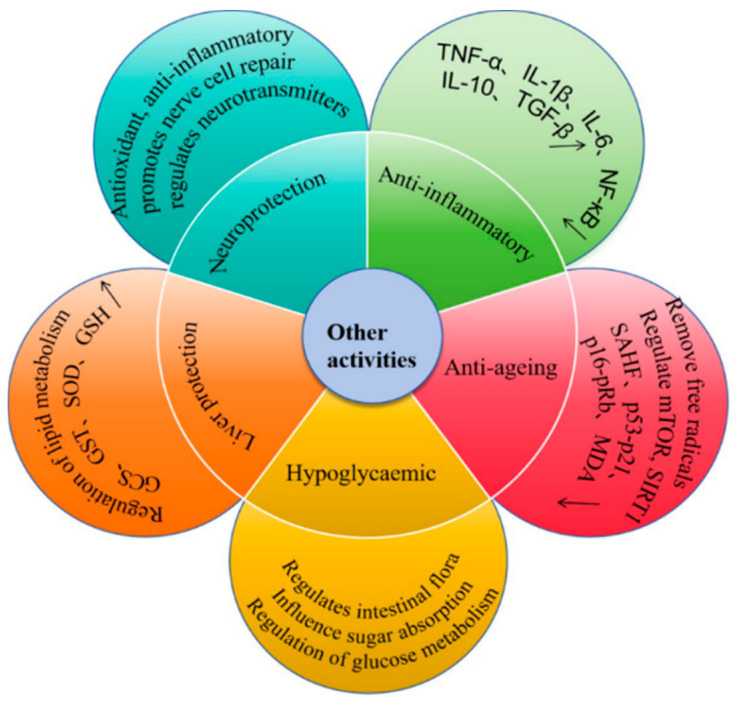
Other activities of polysaccharides. (↑: Indicates upward adjustments or increased secretion, ↓: Indicates downward adjustment or inhibit secretion).

COPs were able to produce a significant inhibitory effect on the proliferation of breast cancer cells MCF-7. This inhibition was not only limited to the progression stage of the cell cycle but also included the migration and invasion ability of the cells. More importantly, COPs seemed to stimulate MCF-7 cells to undergo an apoptotic process, which could effectively inhibit the growth of transplanted tumors in nude mice [97]. COPs could inhibit AGS cell proliferation by ELF3-AS1 as well as promote AGS cell apoptosis [98] and could improve epilepsy (RE) in children. This may be due to the inhibition of MDR1B and MVP mRNA and protein expression [99]. Pushy dogwood polysaccharides may have therapeutic effects on alcoholic fatty-liver disease (AFLD) as APFC-2 significantly inhibited lipid formation both in vitro and in vivo through the activation of hepatic kinase B1 (LKB1), which in turn modulated the adenosine 5′-monophosphate-activated protein kinase (AMPK)-SREBP-1 and AMPK-PPAR-α pathways [51]. *Cornus officinalis*’s total glycosides and polysaccharides improve cardiac function, reduce the area of myocardial infarction, and promote myocardial mitochondrial biosynthesis in rats with AMI, and their protection of mitochondria in cardiomyocytes of rats with AMI may be achieved through the GSK-3β signaling pathway [100]. The hypoglycemic effect of *Cornus officinalis* polysaccharide PFC-3 was assessed in vitro by glucose uptake and consumption assays and found to enhance glucose uptake and observably improve glucose consumption in insulin-resistant HepG2 cells, which were able to significantly reduce fasting glucose levels, glycated hemoglobin levels, amylase activity, and to improve lipid metabolism and hepatic lesions in streptozotocin-induced diabetic rats [57]. Studying the hyperglycemic, hypolipidemic, and antioxidant properties of fractions of *Cornus officinalis* polysaccharide (FCPC) in STZ-induced diabetic rats, it was found that FCPC had significant inhibitory effects on α-amylase and α-glucosidase and insulin sensitization and also improved the glycogen content in the liver, bone, and muscle and the dyslipidemia and oxidative damage induced by STZ [101]. In addition, COPs have anti-inflammatory, antiaging, neuroprotective, and hepatoprotective properties [7] (Figure 6).

## 5. Conclusions and Future Perspectives

**Figure 7:**
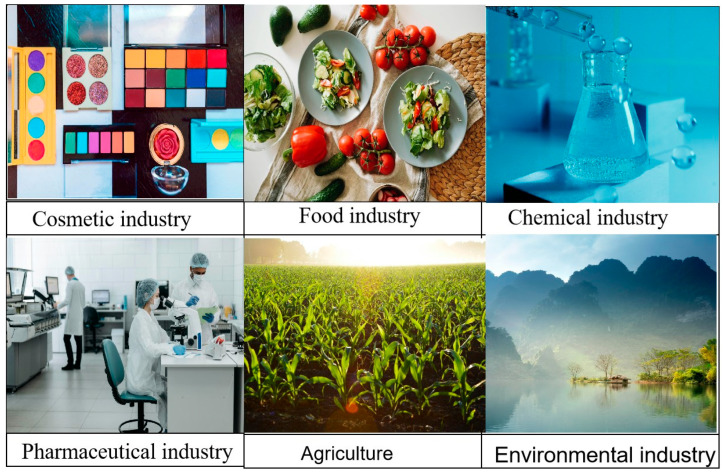
Applications of polysaccharides “https://www.pexels.com/zh-cn/ (2 April 2025)”.

Polysaccharides have achieved numerous research results in recent years due to their non-toxicity, high content, and easy degradation (Figure 7). In this paper, we reviewed several emerging extraction methods of COPs, summarized their purification methods, and described the structural features in terms of monosaccharide composition, average molecular weight, chemical structure, and chemical modification. In the presentation of biological activities, the main focus was on antioxidant, antibacterial, antitumor, and immunomodulatory aspects. Finally, a summary outlook on the polysaccharides of *Cornus officinalis* was presented.

Despite the rich nutritional and medicinal value of *Cornus officinalis*, there have been few achievements in the diversified utilization of *Cornus officinalis*. However, the development of COPs still faces great opportunities and challenges. At the same time, advanced food processing technology is conducive to the development and economic sustainability of the components of *Cornus officinalis*, by continuously improving the diversified utilization of *Cornus officinalis*, increasing the added value of the products, promoting high-quality development in related industries, and laying the foundation for the development of COPs. Firstly, the long production cycle and processing difficulty of *Cornus officinalis* may be the result of a combination of natural properties, technological limitations, and an imperfect industrial chain. In addition, the high production cost is mainly reflected in the high time cost of *Cornus officinalis* powder decontamination; in conventional extraction, hot water extraction requires long times, high-temperature heating, and high energy consumption; ultrasonic and microwave equipment’s initial investment is high, it has a high maintenance cost, and part of the enzyme preparation is difficult to prepare, raising the price. By comparing with other auxiliary extraction methods, the extraction efficiency of hot water extraction is low. Under the same conditions, the low extraction efficiency of COPs may also be caused by the improper handling of raw materials in the experimental process, instrumental parameter settings, and operational errors. The extraction of COPs is mostly carried out by conventional extraction methods, as there are still unknown risks for emerging extraction methods, and the related composite technology lacks systematic optimization. Moreover, we also have to consider the environmental protection and cost constraints. Therefore, the current extraction methods are limited. In conclusion, it is necessary to achieve efficient and sustainable production in the future through Cornelian cherry varieties’ innovation, mechanization upgrading, process standardization, and industry chain development. For the COP extraction process, it is necessary to break through the bottleneck of traditional extraction through technological innovation and process integration to achieve green and efficient production in order to determine the high purity and quality of the product. Secondly, most of the current research on plant polysaccharides focuses on the determination of primary structures, and the determination of advanced structures still requires the development and application of advanced technologies. These techniques can help to further clarify its related complex structure and physicochemical characteristics, further promote the study of the correlation between chemical structure and biological activity, and have important practical applications for clinical research. Thirdly, the application of modified polysaccharides in foodstuffs is less researched and needs to be further developed, as there is less evaluations of the toxicity aspect of polysaccharides after chemical modification, and the use of toxic reagents may result in environmental pollution; the conformational relationship between the modified polysaccharide structure and bioactivity needs further in-depth study [102]. Finally, to improve the commercial use of *Cornus officinalis*, increase the resource utilization of *Cornus officinalis*, and diversify the discovery of related products, for example, polysaccharide research can produce green organic products, deep-processed products, and strengthen the development of related functional factors.

## Figures and Tables

**Table 1:** Regional distribution of *Cornus officinalis* in China (China Species Library—Flora).

Region	Species	Genus	Family	Region	Species	Genus	Family
Yunnan	17	2	1	Jiangsu	6		
Guangxi	12	2		Anhui	6		
Shanxi	10	1		Liaoning	5		
Zhejiang	10			Hebei	4		1
Sichuan	10			Fujian	4		1
Henan	9	1		Taiwan	4		1
Guangdong	9	1		Xizang	5		
Jiangxi	9			Jilin	4		
Gansu	9	1		Neimenggu	3	1	
Guizhou	8			Qinghai	2		
Shanxi	6	1		Heilongjiang	1		
Hunan	7			Xinjiang			1
Hubei	7			Chongqing	1		
Hainan	5	1	1	Aomen	1		
Shandong	6						

**Table 2:** Extraction of traditional polysaccharides.

Traditional Extraction Method	Advantage	Disadvantage	References
Hot water extraction	Easy and low-cost extraction	Low extraction rate	[15,24,25,26,27]
Acid (base) extraction	Applicable to specific polysaccharides	Possible damage to polysaccharide structure, complex processing, high equipment requirements	
Ultrasonic extraction	High extraction rate and simplicity	Glycosidic bond breaking by high ultrasound	
Microwave extraction	Time-saving, efficient, energy-saving	Solvent residue	
Enzyme extraction	Environmentally friendly and efficient	High environmental requirements, not for large-scale industrial production	

**Table 3:** Structural segment of *Cornus officinalis* polysaccharides.

Name	Structural Segment	References
APFC-2	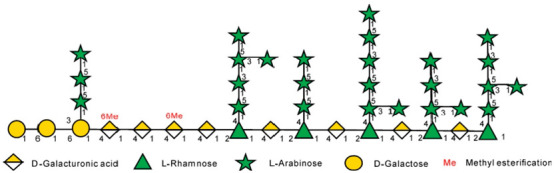	[51]
FCAP1	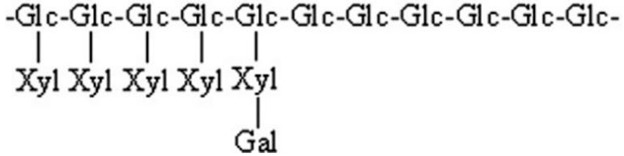	[6]
PFC-3	NM	[57]

NM, not mentioned.

**Table 4:** Chemical modification of other plant polysaccharides.

Resource	Compound Name	Monosaccharide Composition	Molecular Mass (kDa)	Chemical Modification	DS	Effects	Reference
*Zizania latifolia*	ZLP	Ara, Gal, GlcUA, Xyl, GalU, Glc	0.91–29.6	DMF, SA	15.1 ± 2.50	Antioxidant	[65]
*Dioscorea* spp.	CYP	Rha, Gal, GlcA, Xyl, GalA, Glc	333	CSA-Pyr	0.51 ± 0.05	Immunity regulation	[66]
*Taraxacum* *Mongolicum Hand.—Mazz*	DRPs	Man, GlcN, Rha, Glc, Gal, Ara	NM	Concentrated sulfuric acid: n-butanol = 3:1	1.49 ± 0.07	Glucose reduction, probiotic value-added, antioxidant	[67]
*Medicago sativa* L.	AP	Fuc, Ara, Gal, Glc, Xyl, GalU	2.2 × 10^4^	CSA-Pyr	0.724	Antioxidant, antimicrobial	[68]
*Leyss. ex Fr*.	GLP	NM	9.4 × 10^4^–62.7 × 10^4^	SO_3_·Py	0.83–1.74	Bovine intestinal epithelial cell proliferation-promoting, anti-obesity, anticoagulation	[69]
*Ginkgo biloba leaf*	GBLP-3	Man, Rha, Gal, GlcUA, Ara	NM	POCl_3_-Pyr	0.228	Antioxidant	[70]
*Momordica charantia*	Momordica charantia polysaccharide	Rha, GalUA, Gal, Xyl, Ara	NM	DMF, POCl_3_, Pyr	0.12 ± 0.08	Antioxidant	[71]
*Trichosanthes peel*	TPP-1	NM	NM	(NaPO_3_)_3_, Na_5_P_3_O_1_, NaOH	0.43	Antioxidant	[72]
*Garlic*	Garlic polysaccharide	NM	NM	DMF, POCl_3_, C_6_H_15_N	0.04	Antioxidant	[73]
*Pumpkin*	Pumpkin polysaccharide	NM	NM	POCl_3_-Pyr	0.01–0.02	Antioxidant	[74]
*Desmodium styracifolium*	DSP0	Glc, GalUA, Ara, Gal, Rha, Xyl, Man	9.68	C_3_H_8_O, NaOH, ClCH_2_COOH	NM	Antioxidant, repair damaged HK-2 cells, maintain cell physiology	[75]
*Lotus root*	LRP	GalUA, Glc, Gal, Ara, Man, Rib, Rha, GlcUA	NM	NaOH, ClCH_2_COOH	0.309–0.514	Antioxidant	[76]
*Panax japonicus* C. A. Mey	PJPS	Man, D-Rib, GlcUA, Gal, D-GalUA, Glc,	NM	NaOH, ClCH_2_COOH	0.973	Antioxidant	[77]
*Orchis chusua* D. *Don* (*Salep*)	SP	Glu, Man	369	C_4_H_6_O_3_, NaOH, NaHCO_3_, HCI	NM	Probiotic capacity, antioxidant	[78]
*Garlic*	PS	NM	NM	C_4_H_6_O_3_, NaOH, HCI	0.5	Antioxidant	[79]
*Garcinia* *mangostana* L.	UAEE-PMSP	Ara, Rha, GalUA	NM	C_4_H_6_O_3_, NaOH	0.33	Antioxidant	[80]
*Lonicera caerulea* L. fruits	PLP	GalA, Rha, Ara, Man, Glc, Gal	5.9 × 10^4^	HNO_3_-Na_2_SeO_3_	NM	Antioxidant	[81]
*Dandelion roots*	DRP	GalA, Rha, Ara, Man, Glc, Gal	8.7	HNO_3_-Na_2_SeO_3_		Antioxidant, immunomodulation	[82]
*Medicago sativa* L.	RAPS-1 RAPS-2	Rha, Xyl, Ara, GalUA, Man, Glc	10.0, 15.8	HNO_3_-Na_2_SeO_3_		Antioxidant, antitumor	[83]
*Green tea*	TPS-1	Rha, Ara, Gal, Glc, Xyl, Man, Fru, GaIA	NM	HNO_3_-Na_2_SeO_3_		Glycosidase inhibitory activity	[84]

NM, not mentioned.

## Data Availability

No new data were created or analyzed in this study. Data sharing is not applicable to this article.
